# Increased Callosal Thickness in Early Trained Opera Singers

**DOI:** 10.1007/s10548-025-01134-x

**Published:** 2025-08-07

**Authors:** Boris Kleber, C. Dale, A. M. Zamorano, M. Lotze, E. Luders, F. Kurth

**Affiliations:** 1 Center for Music in the Brain (MIB), Department of Clinical Medicine, Aarhus University & Royal Academy of Music Aarhus/Aalborg, Aarhus, Denmark; 2 School of Psychology, University of Auckland, Auckland, New Zealand; 3 Functional Imaging Unit, Department of Diagnostic Radiology and Neuroradiology, University of Greifswald, 17475 Greifswald, Germany; 4 Department of Women’s and Children’s Health, Uppsala University, Uppsala, Sweden; 5 Laboratory of Neuro Imaging, School of Medicine, University of Southern California, Los Angeles, USA; 6 Department of Diagnostic and Interventional Radiology, Jena University Hospital, Jena, Germany

**Keywords:** Corpus callosum, Brain, White matter, MRI, Professional singers

## Abstract

Structural adaptations of the corpus callosum have been well documented in early-trained instrumental musicians, reflecting experience-dependent plasticity in response to bimanual coordination and auditory–motor integration. Although the sensorimotor demands of singing differ, professional vocal training also requires precise control of bilateral vocal tract musculature and integration of auditory feedback; yet, less is known about whether similar adaptations occur in professional singers. This study used structural neuroimaging to investigate variations in callosal thickness in relation to vocal training in 55 participants, including 27 professionally trained opera singers and 28 non-singers. A significant negative correlation between age at first singing lesson and callosal thickness was observed in singers, with effects surviving correction for multiple comparisons in the anterior third (rostrum, genu, rostral body), at the anterior-posterior midbody border, and the isthmus. While group comparisons revealed greater callosal thickness in singers than non-singers in these same regions, these differences did not remain significant after correction. Likewise, a positive correlation between years of professional singing and callosal thickness in the midbody did not survive correction for multiple comparisons. Our main finding aligns with prior evidence of training-related plasticity in the corpus callosum and suggests that early musical experience—including in the context of intensive vocal practice—may contribute to enhanced interhemispheric connectivity. Although the current design does not allow us to isolate effects specific to singing compared to other forms of sensorimotor training, the results underscore developmental timing as a key factor in how prolonged musical experience may shape brain structure.

## Introduction

Professional musicians are well-known for their exceptional motor skills, displayed both visually and acoustically as they excel in playing their instrument. Commencing formal education in early childhood, they dedicate years of practice to developing, perfecting, and maintaining the execution of highly complex motor sequences with millisecond precision (Jabusch et al. 2009; Braun Janzen et al. 2014; Penhune 2022). This extraordinary level of sensorimotor and auditory control has made musicians an ideal model for studying the brain’s capacity for use-dependent plasticity (Jancke 2009; Herholz and Zatorre 2012; Leipold et al. 2021). Among the neural adaptations linked to long-term musical training, structural differences in the corpus callosum (CC)—the brain’s largest interhemispheric fiber bundle—have been consistently reported. Early-trained instrumentalists, in particular, show enhanced callosal structure and connectivity, reflecting increased demands on bimanual coordination and auditory–motor integration (Schlaug et al. 1995; Lee et al. 2003; Gärtner et al. 2013; Steele et al. 2013; Leipold et al. 2021). Yet it remains unclear whether comparable adaptations occur in musicians whose training primarily engages the vocal motor system.

Singing, like instrumental performance, involves long-term auditory–motor training, but relies on distinct effectors and control demands. Unlike instrumentalists, singers cannot depend on visual guidance or externalized motor targets; instead, they must precisely coordinate respiration, phonation, and articulation using internally monitored cues (Sundberg 1987; Mürbe et al. 2004; Welch et al. 2019). This coordination is achieved through a vocal motor system that undergoes protracted maturation across childhood and adolescence (Smith and Zelaznik 2004; Sadagopan and Smith 2008; Ross et al. 2011). To meet the demands of a musical framework, singers must develop exceptional control over tonal, temporal, and timbral dimensions of sound production—skills that far exceed the requirements of speech (Zatorre and Baum 2012; Harris et al. 2023). These heightened demands place unique pressure on the speech motor system, requiring the integration of auditory and somatosensory feedback with finely tuned control of respiratory, laryngeal, and vocal tract musculature (Jurgens 2002; Simonyan and Horwitz 2011; Zuk et al. 2022). Operatic singers, in particular, must maintain expressive vocal control while meeting high technical expectations on sound production, which in turn places unique demands on the neural circuits involved in complex sensorimotor integration and cognitive control (Callan et al. 2006; Kleber et al. 2010, 2013, 2017; Zarate 2013).

Recent work has emphasized how long-term musical training can induce widespread structural changes across sensorimotor, auditory, and associative brain regions, with patterns of volumetric and microstructural reorganization closely aligning with behavioral expertise (Olszewska et al. 2021; Leipold et al. 2021). While these adaptations have been most consistently demonstrated in instrumentalists—particularly in relation to cortical and subcortical motor structures, auditory cortex, and interhemispheric pathways—they overall highlight how specialized occupational training can leave measurable and lasting imprints on brain morphology (Wu et al. 2020). Based on substantial research linking motor precision, feedback-based learning, and auditory-motor integration to neuroplastic changes in instrumental musicians (Schlaug et al. 1995; Munte et al. 2002; Schneider et al. 2002; Lee et al. 2003; Gaser and Schlaug 2003; Bermudez and Zatorre 2005; Jancke 2009; Steele et al. 2013; James et al. 2014; Groussard et al. 2014; Sato et al. 2015; Elmer et al. 2016; Karpati et al. 2017; Olszewska et al. 2021; Leipold et al. 2021; Penhune 2022), we argue that singing—though distinct in its reliance on internalized control and vocal tract coordination—may likewise engage experience-dependent neuroplasticity in key white-matter structures such as the corpus callosum. Yet despite the large interest in vocal motor control, studies investigating structural neural plasticity in trained singers remain sparse (Halwani et al. 2011; Kleber et al. 2016; Wang et al. 2019; Perron et al. 2021).

Considering the unique sensorimotor demands of singing (Harris et al. 2023) and the corpus callosum’s essential function in interhemispheric communication within the speech motor system (Friederici et al. 2007; Kort et al. 2014, 2016; Belyk et al. 2015; Neef et al. 2015), the present study investigates callosal morphology in professionally trained opera singers compared to non-singers. As demonstrated in prior morphometric research, callosal thickness serves as a sensitive indicator of interhemispheric transfer efficiency and plastic changes associated with prolonged training (Luders et al. 2012), providing a validated structural metric for assessing experience-dependent neuroanatomical variation. Based on functional data suggesting that language and vocal-motor tasks modulate interhemispheric interactions via the corpus callosum—particularly in the midbody and isthmus (Karbe et al. 1998), we hypothesize that the complex sensorimotor training required for operatic singing may lead to increased callosal thickness in these subregions. In line with previous findings in early-trained instrumentalists, we further hypothesize that such structural adaptations will be most pronounced in individuals who began singing training earlier in life, due to heightened neuroplastic sensitivity during childhood and adolescence (van der Knaap and van der Ham 2011). Finally, we explore whether accumulated years of professional singing experience are also associated with callosal morphology, as a marker of continued training-related plasticity in adulthood.

## Methods

### Study Sample

We enlisted 55 right-handed individuals with no reported history of neurological or psychiatric disorders. Handedness was determined using the Edinburgh Handedness Inventory (Oldfield 1971). The same cohort was previously included in a voxel-based morphometry (VBM) study of singing-related gray matter differences (Kleber et al. 2016). For the purpose of examining a distinct anatomical structure (corpus callosum) using a different analytic framework, the participants were classified into two cohorts: a group of professional classical singers (*n* = 27; mean age = 26.6 years; age range = 20–34 years, including 19 females) and a control group without formal vocal training (*n* = 28; mean age = 24.9 years; age range = 21–31 years; 21 females).

The singer cohort included members from prominent local musical institutions: two from the Stuttgart State Opera, one from the SWR Radio Vocal Ensemble Stuttgart, and 24 from the State University of Music and Performing Arts Stuttgart. The latter group was composed of students engaged in advanced studies in vocal performance or opera. On average, these singers began their formal training at 15.7 years old (SD = 3.7; range = 7–25 years), accumulating an average of 10.4 years of professional experience (SD = 4.2; range = 3–23 years) by the study’s commencement, and dedicating about 17.1 h per week to singing practice (SD = 6.6; range = 5–30 h).

The control group was recruited from the academic community at the University of Tübingen, primarily from the faculties of psychology and medicine. To maintain comparability with the singer group, controls with modest instrumental training were included, with an average musical practice of 3.4 years (range = 1–8 years), which included basic training in piano (*n* = 6), recorder (*n* = 9), and guitar (*n* = 3), typically acquired during childhood or early adolescence. However, controls with any extensive singing involvement—defined as more than 5 h of singing per week—or any formal vocal training were excluded. Written informed consent was obtained from all subjects prior to participation, in accordance with the Declaration of Helsinki. The institutional ethics review board of the Medical Faculty at the University of Tübingen approved the study protocol.

### Data Acquisition and Preprocessing

Brain images were obtained using a 1.5 T Sonata whole-body scanner (Siemens Medical Systems, Erlangen, Germany) with a whole-body coil for transmission and an 8-channel phased-array head coil for reception. We used a T1-weighted 3D magnetization prepared rapid gradient echo (MPRAGE) sequence with an isotropic spatial resolution of 1 mm^3^. For each participant, 176 slices were acquired with an image matrix size of 256 × 256 and a FOV = 256 mm × 256 mm^2^. Other image acquisition parameters were TR/TE/TI = 1300/3.19/660 ms, bandwidth = 190 Hz/Px, and flip-angle = 15°. Head-motion was minimized during scanning with a rubber-foam restraint. All images underwent a visual quality control by one rater (BK).

All brain images were pre-processed in SPM12 (http://www.fil.ion.ucl.ac.uk/spm) and the CAT12 toolbox (Gaser et al. 2024) applying corrections for magnetic field inhomogeneities and spatial alignment using rigid-body transformations. In addition, the total intracranial volume (TIV) was estimated for each subject by classifying images as gray matter (GM), white matter (WM), and cerebrospinal fluid (CSF) and adding the sub-volumes of these compartments (TIV = GM + WM + CSF).

### Callosal Thickness Estimation

Using the preprocessed images, the corpus callosum was manually outlined by one rater (C.D.) in each brain’s midsagittal section (Luders et al. 2007a). The callosal traces were extracted and automatically processed in a number of successive steps (Luders et al. 2011, 2014, 2018). More specifically, the callosal outlines were separated into 100 nodes and re-sampled at regular intervals, rendering the discrete points comprising the two boundaries spatially uniform. A new midline curve was then created by calculating the two-dimensional average from the corresponding 100 equidistant nodes of the upper and the lower callosal boundaries. Finally, the distances from each of the 100 upper and lower boundary nodes to the corresponding point on the midline were computed and averaged, yielding thickness estimates at 100 equidistant points across the midsagittal callosal surface. This high-resolution, surface-based approach has been validated in previous work and allows for the detection of localized variations in callosal morphology (Luders et al. 2009, 2012).

### Statistical Analyses

**Fig. 1:**
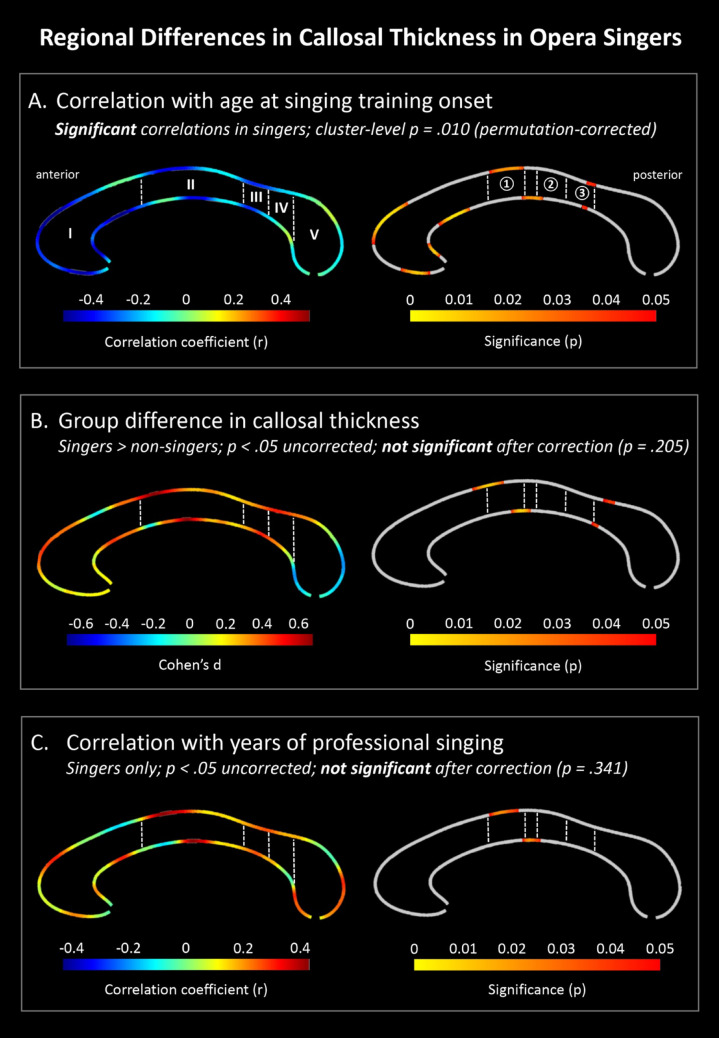
Each subplot displays point-wise statistics along the callosal outline. Color bars show uncorrected p-values (right) and corresponding effect sizes (left; Cohen’s d for group comparisons, Pearson’s r for correlations). Cluster-level corrections for multiple comparisons are reported in the panel descriptions. They were computed using nonparametric permutation testing with 10,000 permutations, as previously established (Thompson et al. 2004; Luders et al. 2009; Anastasopoulou et al. 2017). (**A**) Earlier onset of vocal training in singers was significantly associated with greater callosal thickness (permutation-corrected *p* =.010). Effects were localized to the anterior third (rostrum, genu, rostral body), anterior-posterior midbody border, and isthmus. (**B**) Group differences between singers and non-singers showed greater callosal thickness in singers in overlapping regions, but these differences did not survive correction (permutation-corrected *p* =.205). (**C**) Positive correlations with years of professional singing were also restricted to uncorrected thresholds and did not survive correction (permutation-corrected *p* =.341). Parcellation boundaries mapped on the left callosal outline follow Chao et al. (2009): I – Rostrum, Genu, and Rostral Body; II – Anterior Midbody; III – Posterior Midbody; IV – Isthmus; V – Splenium. Numbers on the right callosal outline indicate the approximate projection zones of ① larynx/tongue, ② head/face, and ③ upper limb primary motor areas, adapted from Domin and Lotze (2019).

The statistical analyses were conducted in Matlab (The MathWorks, Natick, MA) using a mass-univariate general linear model. The calculated point-wise callosal distances constituted the dependent variable, group the independent variable, and age as well as TIV covariates of no interest. In addition to the group comparison (opera singers vs. controls), we conducted two correlation analyses within opera singers, examining (i) the link between callosal thickness and the age at the first singing lesson, and (ii) the link between callosal thickness and the years of professional singing experience. Again, age and TIV were considered covariates of no interest. The color bars in the right section of Fig. 1 encode the p values associated with the statistical tests performed at each distance value at the upper and lower callosal boundaries. In addition, we report effect sizes at each point (Cohen’s d for group comparisons; Pearson’s r for correlations), which are visualized along the left callosal outlines to indicate the magnitude and spatial distribution of the effects. To control for multiple comparisons, we employed nonparametric permutation testing using a Monte Carlo simulation with 10,000 permutations, as previously established (Thompson et al. 2004; Luders et al. 2009; Anastasopoulou et al. 2017). This approach provides a single corrected p-value per analysis, reported in the corresponding subplot headers.

## Results

As shown in Fig. 1 A, the age at first singing lesson in singers showed a significant negative correlation with callosal thickness. Specifically, an earlier onset of vocal training was associated with greater thickness within the anterior third (rostrum, genu, rostral body), at the border between anterior and posterior midbody, and at the isthmus. This effect remained significant after correction for multiple comparisons (permutation-corrected *p* =.010).

Group differences between opera singers and non-singers (Fig. 1B) showed greater callosal thickness in singers at uncorrected levels (*p* <.05) in overlapping regions—at the border between anterior and posterior midbody and the isthmus. However, these differences did not survive correction for multiple comparisons (permutation-corrected *p* =.205). No areas showed significantly greater thickness in controls at any threshold.

With respect to years of professional singing experience (Fig. 1 C), we observed positive correlations with callosal thickness in the border region between anterior and posterior midbody at uncorrected levels (*p* <.05), but these did not survive correction (permutation-corrected *p* =.341).

## Discussion

This study reveals significant neuroplastic adaptations within the corpus callosum in early trained classical singers, indicating that an earlier start to vocal training is associated with greater callosal thickness in regions implicated in executive functions, motor coordination, auditory processing, and sensory integration. While other associations with group status and years of professional singing experience did not survive correction for multiple comparisons, the spatial convergence of effects supports the relevance of these regions for training-related plasticity. These observations suggest that enhanced interhemispheric connectivity may reflect the heightened sensorimotor demands of singing at a professional level compared to speech production. Together, our findings underscore the potential importance of developmental timing in shaping experience-dependent callosal plasticity, extending evidence from instrumental musicians to vocal artists.

### Functional Organization of the Corpus Callosum

The corpus callosum is the largest white matter structure in the brain and facilitates interhemispheric communication by connecting various cortical areas across the left and right cerebral hemispheres mostly in a homotopic fashion (Luders et al. 2018). As such, it has been subdivided into functionally distinct subregions: the *rostrum* connects the fronto-basal cortex, supporting executive functions and emotional regulation; the *genu* links the prefrontal and anterior cingulate cortices, involved in decision-making, attention, and social behavior; the *rostral body* connects the prefrontal cortex and higher-order premotor areas, including parts of the insula; the *anterior midbody* links supplementary motor and premotor areas essential for planning and coordinating complex movements; the *posterior midbody* associates with premotor and partially with primary motor areas; the *isthmus* connects primary motor, sensory, and auditory regions; and finally, the *splenium*, connecting the parietal, temporal, and occipital lobes, plays a key role in integrating sensory information across hemispheres (Witelson 1989; Zarei et al. 2006; Hofer and Frahm 2006; van der Knaap and van der Ham 2011). Their role in the regulation of communication between hemispheres is influenced by callosal fiber composition (size and density), which determines interhemispheric transfer times (Aboitiz et al. 1992a, b; Clarke and Zaidel 1994). The thickness of the corpus callosum (CC) moreover contributes to interhemispheric integration. Thicker callosal regions, particularly in posterior and anterior sections, are associated with greater hemispheric connectivity, facilitating more efficient transfer of information across hemispheres (Luders et al. 2007b, 2012). Notably, the corpus callosum develops and matures throughout childhood and adolescence with increasing myelination, reflecting corresponding changes in cognitive and motor functions (van der Knaap and van der Ham 2011). These anatomical-functional distinctions provide a critical foundation for interpreting the regional patterns of training-related plasticity observed in our singer cohort.

### Neuroplastic Adaptations in the Corpus Callosum of Classical Singers

The specialized motor skills of instrumentalists differ notably from those of singers, who fine-tune their speech motor system to meet the heightened precision demands of music production. While limb motor control in instrumentalists is strongly lateralized, vocal control—particularly of the tongue, pharynx, and larynx in singers is bilaterally organized (Jurgens 2002; Simonyan and Horwitz 2011; Pilurzi et al. 2013). The human larynx motor cortex (LMC) additionally makes direct monosynaptic connections to the brainstem’s nucleus ambiguous, which innervates the intrinsic laryngeal muscles. However, these descending fibers cross incompletely at the medulla oblongata, enabling each hemisphere to influence both vocal folds, albeit with a contralateral predominance (Jurgens 2002; Pilurzi et al. 2013; Simonyan 2014; Simonyan et al. 2016). Given the essential role of the LMC in precise pitch control (Dichter et al. 2018), its bilateral control of the left and right vocal folds likely facilitates efficient interhemispheric communication—an adaptation particularly relevant for the high temporal precision required in vocal fold vibration during pitch production, which, in trained high sopranos, can exceed 1,000 oscillations per second (Adachi and Yu 2005).

Interhemispheric communication also plays a crucial role in coordinating speech production and perception (Friederici et al. 2007; Belyk et al. 2015; Neef et al. 2015). For example, coordinating vocal production with real-time auditory feedback depends on dynamic interhemispheric interactions—where the left hemisphere predominantly supports speech motor planning and execution, while the right motor cortex contributes to modulation and fine-tuning (Kort et al. 2014, 2016). These integrative demands intensify with task complexity, particularly in singing, which recruits more bilateral and right-hemisphere contributions than speech (Callan et al. 2006; Ozdemir et al. 2006). Supporting this, recent findings show the left LMC is predominantly active during speech perception, whereas the right LMC is more engaged in cognitively demanding tasks (Liang et al. 2023), suggesting differential hemispheric contribution to the processing of non-linguistic musical voice stimuli (Leveque et al. 2013). This heightened interhemispheric coordination likely supports the rapid integration of auditory and motor processes required for vocal production within a musical framework—especially given the stringent temporal and timbral constraints imposed by trained singing (Zatorre and Baum 2012; Zatorre 2013; Albouy et al. 2020). In light of these increased sensorimotor demands, we hypothesized that intensive vocal training—particularly when initiated early—may induce structural adaptations in the corpus callosum that support enhanced interhemispheric communication.

Our most robust finding was a significant negative association between callosal thickness and age at first singing lesson in singers, surviving correction for multiple comparisons. Specifically, earlier onset of vocal training was associated with increased thickness in the rostrum, genu, rostral body, anterior midbody, and isthmus—regions broadly implicated in executive function, motor planning, and sensorimotor integration. Notably, the anterior midbody cluster, where the strongest association emerged, overlaps with the projection zone of the larynx and tongue motor representations, as identified in structural connectivity analyses by Domin and Lotze (2019). This finding suggests that the timing of vocal training may be a critical factor in shaping interhemispheric pathways, and aligns with broader evidence that links sensitive developmental windows with heightened neuroplastic potential (van der Knaap and van der Ham 2011; Penhune 2022).

Additional comparisons between professionally trained singers and non-singers revealed greater callosal thickness in singers only at uncorrected significance thresholds, particularly at the anterior–posterior midbody border and isthmus—regions that overlapped with those identified in the age-of-onset analysis. However, they did not yield statistically significant differences in callosal thickness after correction for multiple comparisons. Similarly, uncorrected correlations localized to the anterior midbody were found with years of professional singing experience, yet these correlations did not survive correction for multiple comparisons. Although these findings should be interpreted with caution, the spatial convergence of uncorrected results with permutation-corrected effects reinforces the potential involvement of the anterior midbody in vocal training-related plasticity. In particular, given the associations with primary motor areas representing the tongue and larynx (Domin and Lotze 2019). Notably, the anterior midbody has also been implicated in language learning. For example, Coggins et al. (2004) reported increased anterior midbody thickness in bilingual speakers, likely reflecting enhanced interhemispheric coordination of speech articulation and language switching processes. While the nature of the task—singing versus language switching—differs, both require the integration of vocal motor control with higher-order cognitive functions, including performance monitoring and executive regulation.

Our CC findings align with earlier voxel-based morphometry results from the same participant cohort, which showed experience-dependent increases in gray matter volume within right sensorimotor regions involved in vocal control (Kleber et al. 2016), and with previous fMRI studies from the same cohort demonstrating increased activation of auditory–somatosensory integration regions during singing tasks (Kleber et al. 2007, 2010). Although the VBM and CC structural studies differ in anatomical focus and analytical approach—and were not statistically linked in the current analysis—they jointly imply that early and intensive vocal training fosters structural neuroplasticity across both cortical and interhemispheric pathways. Additional findings from separate cohorts further highlight experience-dependent modulation of auditory–somatosensory–motor integration in trained singers, including the dorsolateral prefrontal cortex, supplementary motor area, and key regions of the salience network (Kleber et al. 2013, 2017). Together, these studies suggest that intensive vocal training can induce structural and functional plasticity across cortical and interhemispheric networks required for complex sensorimotor planning and top-down control processes (Sundberg et al. 1993; Sundberg 1994; Dromey et al. 2015; Scherer et al. 2017; Dichter et al. 2018).

In line with this, recent findings indicate that singing training in individuals with aphasia can induce structural neuroplasticity in the corpus callosum, potentially facilitating enhanced interhemispheric communication essential for coordinated vocal production (Sihvonen et al. 2024). Moreover, studies that link overall musical sophistication (including singing) to variation in white matter microstructure of the arcuate fasciculus (Cui et al. 2024) and the corpus callosum (Mehrabinejad et al. 2021), as well as training-related increases in gray matter volume (Chaddock-Heyman et al. 2021), suggest that individual singing ability—regardless of formal training—may contribute to structural and functional adaptations in neural systems supporting auditory–motor integration. This aligns with resting-state fMRI data, showing that self-reported singing ability—as measured by the singing subscale of the Goldsmiths Musical Sophistication Index (Gold-MSI)—can be associated with enhanced functional connectivity among frontal and occipital regions implicated in vocal motor control (Cui et al. 2023). Beyond musical activities, aerobic exercise has been shown to enhance or preserve callosal integrity across the lifespan, suggesting that sustained sensorimotor or cognitive engagement—regardless of domain—can modulate interhemispheric connectivity (Loprinzi et al. 2020).

### Neuroplastic Adaptations in the Corpus Callosum of Instrumental Musicians

Early magnetic resonance imaging (MRI) studies reported a larger anterior corpus callosum region in professional instrumentalists, particularly those who began training before age 7, suggesting that sensorimotor training during sensitive periods supports callosal maturation (Schlaug et al. 1995; Ozturk et al. 2002; Lee et al. 2003). Subsequent diffusion-weighted imaging (DWI) studies further revealed that early-trained musicians typically exhibit greater interhemispheric connectivity in the isthmus and splenium, followed by the anterior and posterior midbody, with fewer findings in the rostral body and genu (Schmithorst and Wilke 2002; Bengtsson et al. 2005; Imfeld et al. 2009; Steele et al. 2013; Leipold et al. 2021). Moreover, current training intensity in middle-aged musicians appears to influence the isthmus and splenium, linking enhanced interhemispheric communication to improved bimanual fine-motor control (Gärtner et al. 2013). Collectively, these studies underscore the critical role of the corpus callosum in coordinating bilateral brain functions necessary for auditory processing and sensorimotor integration in highly trained instrumentalists (Leipold et al. 2021; Penhune 2022).

### Comparative Insights Between Singers and Instrumentalists

Despite the bilateral cortical representation of phonation-related muscles in singers, our findings suggest both shared and unique patterns of use-dependent plasticity in the corpus callosum as compared to existing reports from instrumental musicians. For instance, greater callosal thickness in the isthmus and at the border between the anterior and posterior midbody observed in our singer cohort aligns with previous findings in early-trained instrumentalists, supporting a domain-general role of these regions in auditory–motor integration and fine motor coordination (Schlaug et al. 1995; Lee et al. 2003; Steele et al. 2013; Leipold et al. 2021). This interpretation is consistent with functional imaging studies showing that expert singers, like instrumentalists, engage sensorimotor circuits in an experience-specific manner (Kleber et al. 2010, 2013, 2016, 2017).

By contrast, increased anterior callosal thickness in singers—specifically in the rostrum, genu, and rostral body, regions associated with prefrontal processing, emotional regulation, and executive functions—is less commonly reported in instrumentalists. While direct comparisons are not possible in the absence of an instrumentalist control group, these findings may reflect the distinct performance demands of singing, which rely on internally guided motor control, expressive intent, and continuous auditory–somatosensory self-monitoring rather than visually directed, externally anchored actions. Such anterior callosal adaptations may therefore reflect the integration of higher-order cognitive–affective processes into vocal motor performance, rather than implying categorical differences in training effects.

Support for this interpretation comes from broader research on interhemispheric dynamics across different domains of expertise, demonstrating that patterns of interhemispheric connectivity and inhibition can also vary across types of instrumentalists. For example, structural enlargements in the corpus callosum are generally associated with reduced interhemispheric inhibition and enhanced processing (Ridding et al. 2000). However, findings reveal stronger left-to-right interhemispheric inhibition (IHI) in string players compared to pianists, whose IHI is comparable to that of non-musicians (Vollmann et al. 2014). In singers, interhemispheric inhibitory motor interactions are more pronounced during singing than in speech or humming tasks (Lo and Fook-Chong 2004), reflecting the refined bilateral control required for vocal performance. Furthermore, studies on bilingual proficiency suggest that complex vocal tasks may enhance interhemispheric connectivity due to increased cognitive demands, as seen in the strengthened connectivity between Brodmann Areas 44 and 45 and their right-hemispheric homologues (Sander et al. 2023). Together, these findings suggest the possibility that shared domain-general demands on auditory–motor integration are supplemented by domain-specific performance requirements, which may differentially engage subregions of the corpus callosum depending on the sensorimotor and cognitive characteristics of the musical task.

## Conclusions

This study provides evidence for experience-dependent structural variation in the corpus callosum associated with intensive vocal training, particularly in relation to the age at which training begins. Increased callosal thickness in regions such as the rostrum, genu, rostral body, anterior-posterior midbody, and isthmus was significantly associated with earlier onset of singing instruction, suggesting that the timing of musical experience may play a key role in shaping interhemispheric pathways. These adaptations may support the integration of sensorimotor, auditory, and cognitive-emotional processes required for complex vocal behaviors such as operatic singing. They complement previous research on callosal plasticity in instrumental musicians and offer insights into how varied forms of musical expertise may shape brain structure over time. However, due to the limited instrumental training in our sample and the absence of a dedicated instrumentalist group, conclusions regarding the specificity of our findings to singing should remain tentative.

As this study underscores the potential influence of sensitive developmental periods on interhemispheric connectivity, future developmental or longitudinal research—particularly in children and adolescents—will be essential to determine whether vocal training during key neurodevelopmental windows leads to lasting structural and functional brain changes.

## Data Availability

The datasets generated during and/or analyzed during the current study are not publicly available due to European GDPR restrictions but are available from the corresponding author on reasonable request.
